# Supplementary material for: Assessment of liquid media requirements for storing and evaluating respiratory cilia motility
Source: PeerJ. 2025 Mar 27;13:e19191. doi: 10.7717/peerj.19191 (PMC11955192; doi:10.7717/peerj.19191)
Supplement: Supplemental Information 3 — Trachea from 225 Mice were harvested, each trachea was divided into 4 samples, each sample was assigned to a different group. Thus, 225 (mice) × 4 (samples per animal) = 900 different samples that were divided into 180 groups for analysis (n = 5 for each group). Room temperature was maintained in an air-conditioned laboratory at 24 °C. All samples were imaged at 37 °C following storage. [file peerj-13-19191-s003.docx]

| Media | +10% FBS | Storage Temp | Time points (mins) | | | | | |
| --- | --- | --- | --- | --- | --- | --- | --- | --- |
|  |  |  | 0 | 30 | 60 | 120 | 360 | 720 |
| Saline | No | 4°C | n=5 | n=5 | n=5 | n=5 | n=5 | n=5 |
| Saline | No | Room Temp | n=5 | n=5 | n=5 | n=5 | n=5 | n=5 |
| DPBS | No | 4°C | n=5 | n=5 | n=5 | n=5 | n=5 | n=5 |
| DPBS | No | Room Temp | n=5 | n=5 | n=5 | n=5 | n=5 | n=5 |
| DPBS | Yes | 4°C | n=5 | n=5 | n=5 | n=5 | n=5 | n=5 |
| DPBS | Yes | Room Temp | n=5 | n=5 | n=5 | n=5 | n=5 | n=5 |
| HBSS | No | 4°C | n=5 | n=5 | n=5 | n=5 | n=5 | n=5 |
| HBSS | No | Room Temp | n=5 | n=5 | n=5 | n=5 | n=5 | n=5 |
| HBSS | Yes | 4°C | n=5 | n=5 | n=5 | n=5 | n=5 | n=5 |
| HBSS | Yes | Room Temp | n=5 | n=5 | n=5 | n=5 | n=5 | n=5 |
| M199 | No | 4°C | n=5 | n=5 | n=5 | n=5 | n=5 | n=5 |
| M199 | No | Room Temp | n=5 | n=5 | n=5 | n=5 | n=5 | n=5 |
| M199 | Yes | 4°C | n=5 | n=5 | n=5 | n=5 | n=5 | n=5 |
| M199 | Yes | Room Temp | n=5 | n=5 | n=5 | n=5 | n=5 | n=5 |
| DMEM | No | 4°C | n=5 | n=5 | n=5 | n=5 | n=5 | n=5 |
| DMEM | No | Room Temp | n=5 | n=5 | n=5 | n=5 | n=5 | n=5 |
| DMEM | Yes | 4°C | n=5 | n=5 | n=5 | n=5 | n=5 | n=5 |
| DMEM | Yes | Room Temp | n=5 | n=5 | n=5 | n=5 | n=5 | n=5 |
| RPMI | No | 4°C | n=5 | n=5 | n=5 | n=5 | n=5 | n=5 |
| RPMI | No | Room Temp | n=5 | n=5 | n=5 | n=5 | n=5 | n=5 |
| RPMI | Yes | 4°C | n=5 | n=5 | n=5 | n=5 | n=5 | n=5 |
| RPMI | Yes | Room Temp | n=5 | n=5 | n=5 | n=5 | n=5 | n=5 |
| MEM | No | 4°C | n=5 | n=5 | n=5 | n=5 | n=5 | n=5 |
| MEM | No | Room Temp | n=5 | n=5 | n=5 | n=5 | n=5 | n=5 |
| MEM | Yes | 4°C | n=5 | n=5 | n=5 | n=5 | n=5 | n=5 |
| MEM | Yes | Room Temp | n=5 | n=5 | n=5 | n=5 | n=5 | n=5 |
| L15 | No | 4°C | n=5 | n=5 | n=5 | n=5 | n=5 | n=5 |
| L15 | No | Room Temp | n=5 | n=5 | n=5 | n=5 | n=5 | n=5 |
| L15 | Yes | 4°C | n=5 | n=5 | n=5 | n=5 | n=5 | n=5 |
| L15 | Yes | Room Temp | n=5 | n=5 | n=5 | n=5 | n=5 | n=5 |

Supplementary Table 2. Overview of the experimental groups used during this study. Trachea from 225 Mice were harvested, each trachea was divided into 4 samples, each sample was assigned to a different group. Thus, 225 (mice) x 4 (samples per animal) = 900 different samples that were divided into 180 groups for analysis (n=5 for each group). Room temperature was maintained in an air-conditioned laboratory at ~24°C. All samples were imaged at 37°C following storage.
